# α-Glucosidase and Pancreatic Lipase Inhibitory Activity of Chemical Constituents from Adzuki Bean (*Vigna angularis*)

**DOI:** 10.3390/life16020235

**Published:** 2026-02-01

**Authors:** Qingfeng Guo, Xia Zhang, Hao Zhang, Mengxue Wang, Haoran Zhou, Meiru Chen, Zhenhua Yin, Juanjuan Zhang, Baocheng Yang, Li Wang, Lin Chen

**Affiliations:** 1 Henan Engineering Research Center of Chemistry and Biology of Medicinal Resources, Henan Comprehensive Utilization of Edible and Medicinal Plant Resources Engineering Technology Research Center, Zhengzhou Key Laboratory of Medicinal Resources Research, Zhengzhou Key Laboratory of Synthetic Biology of Natural Products, Henan Joint International Research Laboratory of Drug Discovery of Small Molecules, Huanghe Science and Technology College, Zhengzhou 450063, China; 2 School of Pharmacy, Henan University, Kaifeng 475004, China

**Keywords:** *Vigna angularis*, adzuki bean, triterpenoid saponin, *α*-glucosidase inhibition, pancreatic lipase

## Abstract

Originating in East Asia, the adzuki bean (*Vigna angularis*) is a diploid crop mainly grown in this region that belongs to the Fabaceae family. As a potential functional food resource with both medicinal and nutritional value, it offers various health benefits. However, research on its chemical constituents, particularly key bioactive components such as triterpenoid saponins and flavonoids, remains relatively limited. In this study, a new triterpenoid saponin, adzukisaponin A (**1**), along with eleven known compounds (**2**–**12**), were isolated from adzuki bean (*V. angularis*). Among them, compounds **3** (yunganoside B1), **6** (3β-acetyl oleanolic acid), and **7** (β-amyrin) are reported from this source for the first time. Saponins **1**–**3** and flavonoid **8** exhibited significant dual inhibitory activity. Notably, saponins **1**–**3** inhibited pancreatic lipase (IC_50_ = 0.11 ± 0.18 to 0.40 ± 0.21 mM) more strongly than the positive control orlistat, while also acting against α-glucosidase (IC_50_ = 0.14 ± 0.11 to 0.23 ± 0.17 mM). Molecular docking supported their binding to the enzymes’ active sites. This study identifies **1**–**3** and **8** as dual α-glucosidase/pancreatic lipase inhibitors, underscoring the potential of adzuki bean as a source of bioactive functional food ingredients.

## 1. Introduction

Obesity, a metabolic disorder influenced by genetic, dietary, and other factors, has become a global public health challenge [1]. It has been linked to a range of serious comorbidities, including hyperlipidemia and type 2 diabetes, and is also implicated in the development of cardiovascular diseases [2]. Current obesity management approaches include lifestyle interventions, pharmacological treatments, and surgery. However, due to aberrant central appetite regulation and metabolic compensatory mechanisms, long-term weight loss maintenance remains difficult [3]. Moreover, existing therapies have limitations and side effects. Natural bioactive components have attracted considerable attention for their safety and efficacy [4].

Inhibiting pancreatic lipase (responsible for 50–70% of dietary fat hydrolysis) and α-glucosidase (involved in carbohydrate digestion and postprandial blood glucose regulation) represents an important strategy for developing anti-obesity and metabolic disorder agents [1,5]. Adzuki bean (*V. angularis*), which originated in China and is widely cultivated and consumed in East Asia, is traditionally used in Chinese medicine for its diuretic and edema-relieving properties [6]. Modern studies have confirmed that its extracts possess anti-obesity [7], lipid-modulating [8], hypoglycemic [9], and hepatorenal protective activities [10,11], likely attributed to flavonoids and saponins. Nevertheless, a comprehensive understanding of the specific compounds responsible is incomplete. For instance, although HPLC-MS analyses have characterized several flavonoids and saponins [12], and recent phytochemical investigations have led to the isolation of new triterpenoid saponin structures [13] as well as unique phenolic pigments [14], systematic efforts to isolate, unequivocally identify, and quantitatively evaluate the key bioactive flavonoids and saponins—particularly in the context of lipase and glucosidase inhibition—are still relatively limited. In this study, one new triterpenoid saponin and eleven known compounds were obtained from the ethanol extract of adzuki beans and structurally characterized. Their ability to inhibit α-glucosidase and pancreatic lipase were evaluated, providing a scientific basis for their potential development as functional food ingredients aimed at obesity prevention.

## 2. Materials and Methods

### 2.1. General Experimental Procedures

Enzyme α-glucosidase (from baker’s yeast, 77.16 U/mg) was purchased from Beijing Solarbio Science & Technology Co., Ltd. (Beijing, China). Porcine pancreatic lipase (30,000 U/g) and 4-nitrophenyl butyrate (pNPB) were supplied by Shanghai Yuanye Bio-Technology Co., Ltd. (Shanghai, China). The substrate 4-nitrophenyl α-D-glucopyranoside (pNPG), along with reference inhibitors acarbose and orlistat, was obtained from Shanghai Aladdin Biochemical Technology Co., Ltd. (Shanghai, China). An Autopol I automatic polarimeter (Rudolph Research Analytical, Hackettstown, NJ, USA) was used to measure optical rotations. UV spectra were acquired on a Nanodrop2000 ultramicro spectrophotometer (Thermo Fisher Scientific, Waltham, MA, USA). Mass spectrometry involved two systems: an Agilent 6120 series LC/MS for low-resolution ESIMS and a Triple TOFT 6600+ LC/MS (AB Sciex, Framingham, MA, USA) for HRESIMS. A Bruker AV-400 spectrometer (Bruker BioSpin, Rheinstetten, Germany) was employed for NMR experiments. Semi-preparative HPLC was conducted on a Calmflowplus system with a YMC Pack ODS-A column (Kyoto, Japan) and a 50D UV–vis detector (Lumiere Tech Ltd., Beijing, China). For column chromatography, Sephadex LH-20 (Amersham Biosciences, Sweden), silica gel (Qingdao Marine Chemical, Qingdao, China), and ODS-C18 (YMC, Kyoto, Japan) were utilized. TLC spots were detected by heating after spraying with 5% H_2_SO_4_–EtOH.

### 2.2. Adzuki Beans Material

Adzuki beans (*Vigna angularis*) originate from Hegang, Heilongjiang Province, China, and were purchased from Luxianda Biotechnology Co., Ltd. in Jining, Shandong Province, China.

### 2.3. Extraction and Isolation

Adzuki beans (30 kg) were pulverized and extracted three times with 70% ethanol. The combined extracts were concentrated under reduced pressure to afford approximately 1 kg of crude extract. This extract was suspended in water and sequentially partitioned with petroleum ether (PE), ethyl acetate (EtOAc), and n-butanol. Each solvent layer was concentrated and dried to yield the corresponding PE, EtOAc, and n-butanol fractions.

The EtOAc-soluble fraction (47 g) was subjected to silica gel column chromatography, using a gradient elution of CH_2_Cl_2_-MeOH (from 100:1 to 5:1, *v*/*v*), to give eight major fractions (Fr. E1–E8). Subsequent processing of Fr. E2 involved separation over Sephadex LH-20 (CH_2_Cl_2_-MeOH, 1:1, *v*/*v*), which yielded three subfractions (E2-1–E2-3). Recrystallization of E2-1 provided compound **7** (25 mg). In parallel, isocratic chromatography of Fr. E3 on silica gel (PE-EtOAc, 5:1, *v*/*v*) gave compound **6** (38 mg).

The n-butanol fraction (206 g) was enriched on an AB-8 macroporous resin column with stepwise elution (0–95% aqueous ethanol), yielding five fractions (A–E). Separation of Fraction C was carried out on a silica gel column using a stepwise CH_2_Cl_2_-MeOH-H_2_O gradient (9:2:0.1 → 8:2:0.2 → 7:3:0.5, *v*/*v*/*v*). TLC-guided combination of eluates gave four subfractions (C1–C4). C1 was purified by repeated methanol recrystallization to give a white crystalline compound **8** (1.4 g). C2 was purified over Sephadex LH-20 (CH_2_Cl_2_-MeOH, 1:1, *v*/*v*) to yield compound **12** (17 mg). C3 was sequentially chromatographed over Sephadex LH-20 (CH_2_Cl_2_-MeOH, 1:1, *v*/*v*) and silica gel (CH_2_Cl_2_-MeOH, 10:1, *v*/*v*) to afford compounds **9** (178 mg) and **10** (17 mg). Compound **11** (12 mg) was obtained from fraction C4 by purification via semi-preparative HPLC (MeOH-H_2_O, 40:60, *v*/*v*; flow rate: 3 mL/min).

Fraction D was separated by silica gel column chromatography using a stepwise CH_2_Cl_2_-MeOH-H_2_O gradient (9:2:0.1 → 8:2:0.2 → 7:3:0.5, *v*/*v*/*v*), yielding five subfractions (D1–D5) after TLC-based combination. D2 was further purified over Sephadex LH-20 (MeOH-H_2_O, 1:1, *v*/*v*), and the fourth eluted fraction was subjected to semi-prep HPLC (MeOH-H_2_O, 68:32, *v*/*v*; 3 mL/min) to afford compounds **4** (2.6 mg, *t*_R_ = 35.9 min) and **5** (15.7 mg, *t*_R_ = 49.4 min). D3 was directly purified by semi-prep HPLC (MeOH-H_2_O, 70:30, *v*/*v*; 3 mL/min) to give compound **3** (8 mg, *t*_R_ = 27.8 min). D5 was separated by semi-prep HPLC (CH_3_CN-H_2_O, 72:28, *v*/*v*; 2.5 mL/min) to yield compounds **1** (16 mg, *t*_R_ = 39.5 min) and **2** (24 mg, *t*_R_ = 42.7 min).

Adzukisaponin A (**1**): White amorphous powder; [α]^25^D = +54.8 (c 0.02, MeOH); UV (MeOH) λmax (log ε) 209 (0.16), 212 (0.11), 229 (0.10) nm (Figure S11); ^1^H and ^13^C NMR, see Table 1; Negative ESIMS *m*/*z* 957.4 [M–H]^−^ (Figure S10).

### 2.4. Acid Hydrolysis and GC Analysis of the Sugar Moieties in **1**

Compound **1** (3 mg) was dissolved in 2 M HCl (4 mL) and heated at 95 °C for 10 h. The reaction mixture was evaporated under vacuum, and the residue was extracted three times with CH_2_Cl_2_. Following concentration, the aqueous eluate afforded a residue, which was then subjected to treatment with L-cysteine methyl ester hydrochloride (2.0 mg) in pyridine (1.0 mL) at 60 °C for 2 h. After drying under a stream of N_2_ gas, the residue was reacted with N-(trimethylsilyl) imidazole (0.2 mL) at 60 °C for 1 h. The reaction was worked up by quenching with water (0.5 mL) and extracting with cyclohexane (0.5 mL × 3). The organic phases were combined and dried down to a volume of 1.0 mL, and the resulting solution was submitted for GC analysis [15]. The retention time comparison between the trimethylsilylated derivatives of the sample and those of authentic standards (derivatized in the same manner) served to identify each sugar unit [16]. A GC-2010 Plus gas chromatograph (Shimadzu, Kyoto, Japan) was used for the analysis. The separation was performed on an HP-5MS column (30 m, 0.25 mm I.D., Agilent, Santa Clara, CA, USA); column temperature was 100 °C for 2 min., followed by a gradient of 15 °C/min. to 260 °C. The retention times of the monosaccharide derivatives were as follows: D-Glc, 15.05 min, and D-GlcA, 13.80 min. The retention times of the standard monosaccharide derivatives were: D-Glc, 15.06 min, and D-GlcA, 13.81 min.

### 2.5. Measurement of α-Glucosidase Inhibition

The inhibitory activity was assessed using the PNPG method as described in the supporting information and reference [17].

### 2.6. Measurement of Pancreatic Lipase Inhibition

The inhibition was determined spectrophotometrically using p-nitrophenyl butyrate as substrate, following the procedure in the supporting information and reference [18].

### 2.7. Molecular Docking Simulation

Docking studies were performed to explore the binding modes of the compounds, according to the protocol in the supporting information and reference [19].

### 2.8. Statistical Analysis

All biological experiments were carried out in triplicate to guarantee the reproducibility of the results. The half-maximal inhibitory concentration (IC_50_) values are expressed as the mean ± standard error of the mean (S.E.M.), with statistical computations implemented via GraphPad Prism 10.1.2 software.

## 3. Results and Discussion

### 3.1. Isolation and Structural Elucidation

**Figure 1:**
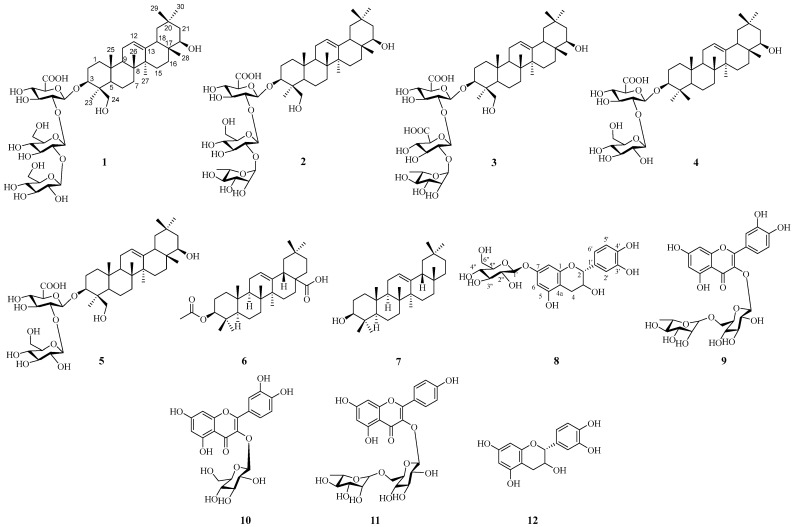
Structure of compounds **1**–**12** isolated from adzuki bean (*V. angularis*). The compounds are: **1** (adzukisaponin A), **2** (azukisaponin V), **3** (yunganoside B1), **4** (azukisaponin I), **5** (azukisaponin II), **6** (3β-acetyl oleanolic acid), **7** (β-amyrin), **8** ((+)-catechin 7-O-β-D-glucopyranoside), **9** (rutin), **10** (isoquercitrin), **11** (kaempferol 3-O-rutinoside), and **12** ((+)-catechin). The numbers and letters represent the standard atom/site numbering system for triterpenoid aglycones and flavonoid glycosides, respectively, following IUPAC nomenclature.

A systematic separation strategy involving silica gel column chromatography, Sephadex LH-20 gel filtration, recrystallization, and semi-preparative HPLC was applied to the 70% ethanol extract of adzuki beans, which afforded 12 compounds (Figure 1). The structure of the novel compound **1** was elucidated via comprehensive NMR spectroscopic analysis (Figures S1–S8), supplemented by HR-ESI-MS (Figure S9) and acid hydrolysis to identify its sugar moiety. Compounds **2**–**12** were identified as known structures by matching their observed physicochemical characteristics and spectral data with existing literature reports.

**Figure 2:**
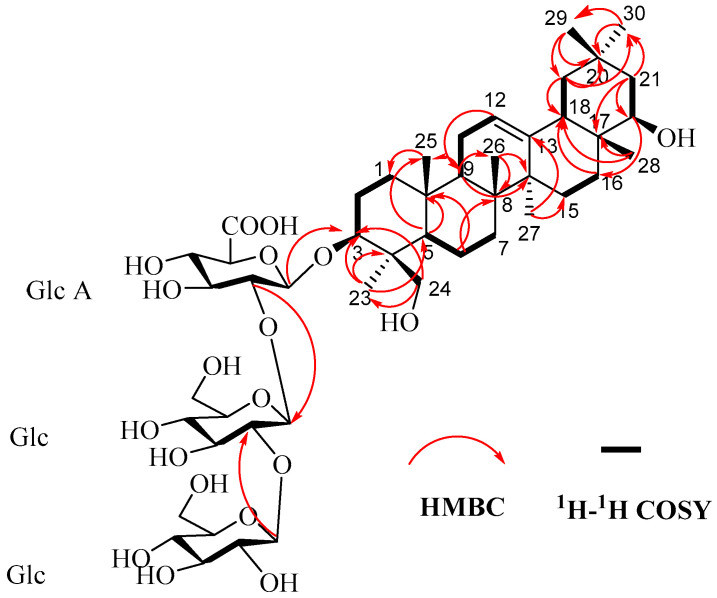
^1^H-^1^H COSY, selected HMBC correlations of compound **1**.

Compound **1** was obtained as a white amorphous powder with [α]^25^D = +54.8 (c 0.02, MeOH). HR-ESI-MS analysis revealed an [M–H]^−^ ion at *m*/*z* 957.5222 (calcd. for 957.5059), suggesting the molecular formula C_48_H_78_O_19_ with an index of hydrogen deficiency (IHD) of 10. The ^1^H-NMR spectrum (Figure S1) showed seven angular methyl singlets at *δ*_H_ 0.69, 0.92, 0.96, 1.20, 1.24, 1.27, and 1.36; one olefinic methine signal at *δ*_H_ 5.27 (br t-like); and three anomeric proton signals at *δ*_H_ 5.08 (1 H, J = 7 Hz), 5.24 (1 H, J = 7.4 Hz), and 5.58 (1 H, J = 7 Hz). The ^13^C-NMR spectrum (Figure S2), exhibited a carbonyl signal at *δ*_C_ 172.2 and two olefinic carbon signals at *δ*_C_ 122.5 and 144.6. These spectroscopic data suggested that compound **1** is an oleanane-type triterpenoid saponin. The signal at *δ*_C_ 90.2 for C-3 indicated glycosylation at this position. Three anomeric carbon signals at *δ*_C_ 103.3, 104.3, and 106.4 further suggested that **1** is a trisaccharide glycoside of an oleanane-type aglycone linked at C-3. The aglycone proton and carbon signals (Table 1) were fully assigned using distortionless enhancement by polarization transfer (DEPT) spectroscopy (Figure S3), homonuclear correlation spectroscopy (^1^H-^1^H COSY) (Figure S4), heteronuclear single-quantum coherence spectroscopy (HSQC) (Figure S5), heteronuclear multiple-bond correlation spectroscopy (HMBC) (Figure S6), nuclear Overhauser effect spectroscopy (NOESY) (Figure S8), and total correlation spectroscopy (TOCSY) (Figure S7). Consistent with the NMR data reported for Soyasaponin I/V and Azukisaponin II/V, the aglycone moiety was therefore assigned as Soyasapogenol B [20]. Acid hydrolysis followed by gas chromatographic analysis of the corresponding trimethylsilylated L-cysteine derivatives, compared with standard samples, identified the three sugar units as two D-glucoses and one D-glucuronic acid (Figure S13). The large anomeric proton coupling constants (J = 7–9 Hz) observed after hydrolysis indicated *β*-configurations for all sugars. The connectivity and sequence of the sugar chain were determined through analysis of 2D NMR data. Key evidence included the HMBC correlation between GlcA H-1 (δ_H_ 5.08) and the aglycone C-3 (δ_C_ 90.2), the HMBC correlation between Glc‴ H-1 (δ_H_ 5.24) and Glc″ C-2 (δ_C_ 85.5), and the ^1^H-^1^H COSY correlation between Glc″ H-1 (δ_H_ 5.58) and H-2 (δ_H_ 4.16) (Figure 2). Therefore, the structure of compound **1** was determined to be 3-O-[β-D-glucopyranosyl-(1 → 2)-β-D-glucopyranosyl-(1 → 2)-β-D-glucuronopyranosyl] 3β,22β,24-trihydroxyolean-12-ene (**1**).

The other compounds were identified as azukisaponin V(**2**) [21], Yunganoside B1 (**3**) [22], azukisaponin I (**4**) [20], azukisaponin II (**5**) [23], 3β-acetyl oleanolic acid (**6**) [24], β-amyrin (**7**) [25], (+)-catechin 7-O-β-D-glucopyranoside (**8**) [26], Rutin (**9**) [27], isoquercitrin (**10**) [28], kaempferol 3-o-rutinoside (**11**) [29], and (+)- catechin (**12**) [30]. The structures of all isolated compounds (**1**–**12**) are shown in Figure 1.

### 3.2. Inhibitory Activity of α-Glucosidase and Pancreatic Lipase

The inhibition activity tests against α-glucosidase and pancreatic lipase indicated that all tested compounds **(1**–**3**, **6**–**12)** exhibited significant inhibitory effects on both enzymes, demonstrating clear structure–activity relationships (Table 2). This finding aligns with prior reports on the enzyme-inhibitory potential of adzuki bean extracts [8]. In the α-glucosidase inhibition assay, IC_50_ values for the isolated compounds ranged from 0.1 to 2.5 mM. The triterpenoid saponins (**1**–**3**) exhibited the strongest activity within the series, consistent with the reported potency of saponin-rich fractions from adzuki beans [8]. Their potent activity aligns with the established critical role of the C-3 glycosyl moiety in oleanane-type saponins for α-glucosidase inhibition, as evidenced by the strong activity (IC_50_ 18.7–154.3 µM, superior to acarbose) reported for ligushicosides A–E from *Ligulariopsis shichuana* [31]. Notably, the flavonoid compounds **8**, **9**, and **12** also showed considerable inhibitory potency, with their activities statistically comparable to those of saponins **1**–**3** (Table 2, sharing superscript ‘a’). This is consistent with the reported structure–activity relationship that flavonols (e.g., compounds **8** and **9**) generally exhibit stronger α-glucosidase inhibition than flavanones (e.g., **12**) and that hydroxylation on the B-ring is a key activity-enhancing feature [32]. In contrast, the remaining flavonoids and triterpenoids (compounds **6**, **7**, **10**, and **11**) were significantly less active (*p* < 0.05). All tested compounds were, however, significantly less potent than the positive control acarbose (IC_50_ = 0.026 ± 0.05 mM) (*p* < 0.05), a trend previously noted for adzuki bean-derived flavonoids [33]. The strong efficacy of saponins is further supported by computational studies predicting high binding affinity for similar compounds, such as adzukisaponin VI and IV, towards α-glucosidase [34]. In the pancreatic lipase inhibition assay, a more pronounced effect was observed. Triterpenoid saponins (**1**–**3**) along with flavonoids **8** and **11** not only showed superior activity to triterpenoids but were also stronger than the positive control orlistat (*p* < 0.05, Table 2, group ‘b’ vs. ‘c’). This highlights their notable advantage against this target, and extends previous reports on the lipase-inhibitory activity of adzuki bean saponins [8]. The remaining compounds (**6**, **7**, **9**, **10**, and **12**) exhibited activity comparable to or slightly weaker than orlistat. Activity data for compounds **4** and **5** are not reported due to insufficient sample quantity and chemical instability, respectively.

### 3.3. Molecular Docking Results

Molecular docking analysis was performed to provide a structural rationale for the observed inhibitory activities. As shown in Table 3, the binding energies of compounds **1**–**3** and **6**–**12** with both α-glucosidase and pancreatic lipase were below −4 kcal/mol, supporting their potential for direct interaction. Notably, the docking results strongly corroborated the structure–activity relationships observed in vitro. The most potent inhibitors identified in the enzymatic assays—triterpenoid saponins **1**–**3** and flavonoid **8**—consistently yielded the most favorable (most negative) docking scores for both targets. Specifically, saponin **1** demonstrated a predicted binding affinity substantially stronger than that of the drug acarbose (−8.2 vs. −6.2 kcal/mol) against α-glucosidase and superior to orlistat (−8.8 vs. −6.8 kcal/mol) against pancreatic lipase. This computational evidence directly aligns with its exceptional experimental potency. These findings are consistent with prior in silico studies on adzuki bean saponins, which also predicted strong binding to carbohydrate-digesting enzymes [34]. Our results extend this understanding, demonstrating that specific triterpenoid saponins and flavonoids can achieve predicted binding modes that outperform standard inhibitors, providing a plausible mechanistic basis for their significant in vitro efficacy.

## 4. Conclusions

This study systematically isolated and identified **12** compounds from the adzuki bean (*Vigna angularis*), including triterpenoid saponins, flavonoids, and triterpenes, with one being a novel compound and three reported from this source for the first time. Bioactivity evaluation revealed that the triterpenoid saponins (**1**–**3**) acted as potent dual inhibitors of α-glucosidase and pancreatic lipase. Notably, their inhibitory activity against pancreatic lipase surpassed that of the drug orlistat. Molecular docking further supported these findings, revealing strong binding interactions with the enzymes’ active sites, which correlates with the observed inhibitory potency.

These results underscore adzuki bean as a promising natural source of dual-enzyme inhibitors and provide a chemical and mechanistic basis for its potential use in functional foods aimed at metabolic health. The identified triterpenoid saponins represent compelling lead compounds for further development, pending future validation in cellular and in vivo models.

## Figures and Tables

**Table 1:** ^1^H (400 MHz) and ^13^C (100 MHz) NMR data for compound **1** in C_5_D_5_N.

NO	H	C	NO	H	C
1	1.31 (m), 0.74 (m)	38.3	25	0.69 (s)	15.4
2	2.30 (d, J = 10.8), 1.93 (m)	26.4	26	0.92	16.7
3	3.44 (d, J = 7.9)	90.2	27	1.24 (s)	25.5
4	-	43.4	28	1.20 (s)	20.9
5	0.81 (m)	55.8	29	0.96 (s)	33.1
6	1.52 (m), 1.21 (m)	18.3	30	1.27 (s)	28.4
7	1.43 (m), 1.25 (m)	32.9	1′	5.08 (d, J = 7.0)	104.3
8	-	39.6	2′	4.10 (m)	82.2
9	1.51 (o), 1.13(o)	47.4	3′	4.71 (m)	77.2
10	-	36.2	4′	4.56 (m)	72.1
11	1.77 (m)	23.7	5′	4.67 (o)	77.6
12	5.27 (t-like)	122.1	6′		172.2
13	-	144.6	1″	5.58 (d, J = 7.0)	103.3
14	-	42.1	2″	4.16 (m)	85.5
15	1.81 (m), 0.99 (m)	26.2	3″	4.27 (m)	76.9
16	1.87 (m), 1.30 (m)	28.4	4″	4.43	69.1
17	-	37.7	5″	3.64 (o)	77.8
18	2.37(d, J = 13.2)	45.0	6″	4.40 (m), 4.29 (m)	60.8
19	1.89 (m), 1.12 (m)	46.5	1‴	5.24 (d, J = 7.4)	106.4
20	-	30.7	2‴	4.06 (o)	76.7
21	1.72 (m), 1.62 (m)	42.1	3‴	4.17	77.3
22	3.71 (m)	75.3	4‴	4.07 (o)	71.1
23	1.36 (s)	22.4	5‴	3.96 (o)	79.2
24	4.33 (d, J = 11.9), 3.35 (d, J = 11.1)	63.2	6‴	4.61(m), 4.26 (m)	62.5

**Table 2:** Inhibition activity of isolated compounds **1**–**3** and **6**–**12** against α-glucosidase and pancreatic lipase **1**–**3** and **6**–**12**.

Compounds	IC_50_ (mM) ± SEM/% Inhibition	
	α-Glucosidase	Pancreatic Lipase
**1**	0.14 ± 0.11 ^a^	0.40 ± 0.21 ^b^
**2**	0.17 ± 0.21 ^a^	0.17 ± 0.21 ^b^
**3**	0.23 ± 0.17 ^a^	0.11 ± 0.18 ^b^
**6**	2.47 ± 0.25 ^c^	2.8 ± 0.27 ^c^
**7**	1.19 ± 0.12 ^c^	1.35 ± 0.19 ^c^
**8**	0.43 ± 0.06 ^a^	0.41 ± 0.07 ^b^
**9**	0.35 ± 0.08 ^a^	1.29 ± 0.11 ^c^
**10**	1.20 ± 0.31 ^c^	0.77 ± 0.06 ^c^
**11**	0.95 ± 0.12 ^c^	0.39 ± 0.04 ^b^
**1** **2**	0.56 ± 0.11 ^a^	1.25 ± 0.17 ^c^
Acarbose	0.026 ± 0.05 ^d^	/
Orlistat	/	1.02 ± 0.25 ^c^

Note: Within each column, values with different lowercase superscript letters (a, b, c, d) are significantly different (*p* < 0.05)

**Table 3:** Binding affinities of compounds **1**–**3** and **6**–**12** against α-glucosidase and pancreatic lipase.

Compounds	Binding Affinities (kcal/mol)	
	α-Glucosidase	Pancreatic Lipase
**1**	−8.2	−8.8
**2**	−7.4	−7.4
**3**	−7.2	−7.5
**6**	−5.6	−5.2
**7**	−4.7	−4.0
**8**	−7.0	−6.9
**9**	−6.5	−5.5
**10**	−5.7	−6.4
**11**	−6.2	−5.1
**12**	−5.9	−6.5
Acarbose	−6.2	/
Orlistat	/	−6.8

## Data Availability

The original contributions presented in this study are included in the article/Supplementary Material. Further inquiries can be directed to the corresponding author.

## Supplementary Materials

The following supporting information can be downloaded at https://www.mdpi.com/article/10.3390/life16020235/s1: Figure S1. ^1^H NMR spectrum of compound **1** in C_5_D_5_N (400 MHz); Figure S2. ^13^C NMR spectrum of compound **1** in C_5_D_5_N (100 MHz); Figure S3. DEPT spectrum of compound **1** in C_5_D_5_N (400 MHz); Figure S4. COSY spectrum of compound **1** in C_5_D_5_N (400 MHz); Figure S5. HSQC spectrum of compound 1 in C_5_D_5_N (400 MHz); Figure S6. HMBC spectrum of compound **1** in C_5_D_5_N (400 MHz); Figure S7. TOCSY spectrum of compound **1** in C_5_D_5_N (400 MHz); Figure S8. NOESY spectrum of compound **1** in C_5_D_5_N (400 MHz); Figure S9. HRESIMS spectrum of compound **1**; Figure S10. EIMS spectrum of Compound **1**; Figure S11. UV spectrum of Compound **1**; Figure S12. Comparison of the acid hydrolysis products of compound **1** with glucose and glucuronic acid by GC.
