# Supplementary material for: α-Glucosidase and Pancreatic Lipase Inhibitory Activity of Chemical Constituents from Adzuki Bean (Vigna angularis)
Source: Life (Basel). 2026 Feb 1;16(2):235. doi: 10.3390/life16020235 (PMC12941854; doi:10.3390/life16020235)
Supplement: Supplementary file 1 [file life-16-00235-s001.zip › life-4097736-supplementary files.pdf]

SUPPLEMENTARY MATERIAL

α -Glucosidase and Pancreatic Lipase Inhibitory Activity of Chemical Constituents from Adzuki Bean (*Vigna angularis*)

Qingfeng Guo [1], Xia Zhang [1,2], Hao Zhang [1,2], Mengxue Wang [1], Haoran Zhou [1], Meiru Chen [1], Zhenhua Yin [1], Juanjuan Zhang [1], Baocheng Yang [1], Li Wang [1] and Lin Chen [1,*]

1 Henan Engineering Research Center of Chemistry and Biology of Medicinal Resources, Henan Comprehensive Utilization of Edible and Medicinal Plant Resources Engineering Technology Research Center, Zhengzhou Key Laboratory of Medicinal Resources Research, Zhengzhou Key Laboratory of Synthetic Biology of Natural Products, Henan Joint International Research Laboratory of Drug Dis-covery of Small Molecules, Huanghe Science and Technology College, Zhengzhou 450063, China

2 School of Pharmacy, Henan University, Kaifeng 475004, China

* Correspondence: lchenchina@163.com

2. Experimental

2.5. Assay of α-Glucosidase Inhibitory Activity

The α-glucosidase inhibitory activity was determined using a 96-well microplate assay, based on a previously described method (Kang et al., 2011) with slight modifications. Briefly, 8 μL of the test sample solution (dissolved and serially diluted in DMSO to concentrations of 1, 0.5, 0.25, 0.125, and 0.0625 mg/mL) was mixed with 112 μL of phosphate buffer (0.1 M, pH 6.8) and 20 μL of α-glucosidase solution (2.5 U/mL , prepared from enzyme with a specific activity of 77.16 U/mg) in a well. The mixture was incubated at 37°C for 15 min. Subsequently, 20 μL of 2.5 mM p-nitrophenyl-α-D-glucopyranoside (PNPG) solution was added to initiate the reaction, followed by another incubation at 37°C for 15 min. The reaction was terminated by adding 80 μL of 0.2 M sodium carbonate (Na_2CO_3) solution to each well. The absorbance was immediately measured at 405 nm using a microplate reader. All assays were performed in triplicate with three parallel wells for each group to ensure reliability and reproducibility. The inhibitory rate was calculated using the following formula, and the IC_{50} value was determined.

Inhibitory rate (%) = $[1-(A_4-A_3)/(A_2-A_1)] \times 100$

In the formula: A_4 is the absorbance of the sample group (8 μL sample + 20 μL α-glucosidase + 112 μL buffer + 20 μL PNPG); A_3 is the absorbance of the sample blank group (8 μL sample + 152 μL buffer); A_2 is the absorbance of the negative control group (8 μL DMSO + 20 μL α-glucosidase + 112 μL buffer + 20 μL PNPG); A_1 is the absorbance of the blank group (8 μL DMSO + 152 μL buffer).

2.6. Assay of Pancreatic Lipase Inhibitory Activity

A modified version of the 96-well microplate method described by Kim et al. (2010) was used to assess the inhibitory activity against pancreatic lipase. Briefly, 50 μL of the sample solution (dissolved and serially diluted in DMSO to concentrations of 2, 0.5, 0.25, 0.125, 0.0625, and 0.03125 mg/mL) and 50 μL of the pancreatic lipase solution (25 U/mL, prepared in 13 mmol/L Tris-HCl buffer, pH 8.0, and centrifuged at 12,000 r/min for 20 min at 4°C) were mixed in a well and pre-incubated at 37 °C for 10 min. Then, 50 μL of the PNPB solution (1 mg/mL, prepared in 13 mmol/L Tris-HCl buffer) was added to initiate the enzymatic reaction. After incubation at 37°C for 20 min, the absorbance was measured at 405 nm. The assay was performed in quadruplicate with three parallel wells per group. The inhibitory rate and IC_{50} value were calculated.

Inhibitory rate (%) = $[[1-(A_d - A_c)/(A_b - A_a)] \times 100$

In the formula: A_d is the absorbance of the sample group (sample + pancreatic lipase + PNPB); A_c is the absorbance of the sample blank group (sample + buffer solution + PNPB); A_b is the absorbance of the negative control group (buffer solution + pancreatic lipase + PNPB); A_a is the absorbance of the blank group (buffer solution + PNPB).

3.7. Molecular Docking Study

Molecular docking simulations were performed to predict the binding affinity and interaction modes of the compounds with the target enzymes (α-glucosidase and pancreatic lipase). Lower binding energy indicates a more stable and favorable interaction.The 3D crystal structures of α-glucosidase (PDB ID: 3A4A) and pancreatic lipase (PDB ID: 1GPL) were obtained from the RCSB Protein Data Bank (http://www.rcsb.org/). Using PyMOL, water molecules and native ligands were removed. The proteins were then prepared with AutoDock Tools 1.5.6 (adding polar hydrogens, assigning Gasteiger charges) and saved in pdbqt format.The 3D structures of the flavonoid compounds were sourced from the TCMSP database (mol2 format). The positive controls, acarbose and orlistat, were downloaded from PubChem (https://pubchem.ncbi.nlm.nih.gov/) in SDF format, converted to 3D structures using Chem3D, and saved as mol2 files. All ligands were subsequently prepared with AutoDock Tools 1.5.6 and converted to pdbqt format. Docking was performed using AutoDock Tools 1.5.6. For each ligand-receptor pair, the conformation with the lowest binding energy was selected, saved as pdbqt, and converted to PDB format using OpenBabelGUI 2.4.1. The interaction patterns (e.g., hydrogen bonds, hydrophobic interactions) within the optimal poses were visualized and analyzed using PyMOL. (Ren et al. 2024)

Table of contents

Figure S1. ^{1}H NMR spectrum of compound **1** in C_5D_5N (400 MHz)

Figure S2. ^{13}C NMR spectrum of compound **1** in C_5D_5N (100 MHz)

Figure S3. DEPT spectrum of compound **1** in C_5D_5N (400 MHz)

Figure S4. COSY spectrum of compound **1** in C_5D_5N (400 MHz)

Figure S5. HSQC spectrum of compound **1** in C_5D_5N (400 MHz)

Figure S6. HMBC spectrum of compound **1** in C_5D_5N (400 MHz)

Figure S7. TOCSY spectrum of compound **1** in C_5D_5N (400 MHz)

Figure S8. NOESY spectrum of compound **1** in C_5D_5N (400 MHz)

Figure S9. HRESIMS spectrum of compound **1**

Figure S10. EIMS spectrum of Compound **1**

Figure S11. UV spectrum of Compound **1**

Figure S12. Comparison of the acid hydrolysis products of compound **1** with glucose and glucuronic acid by GC

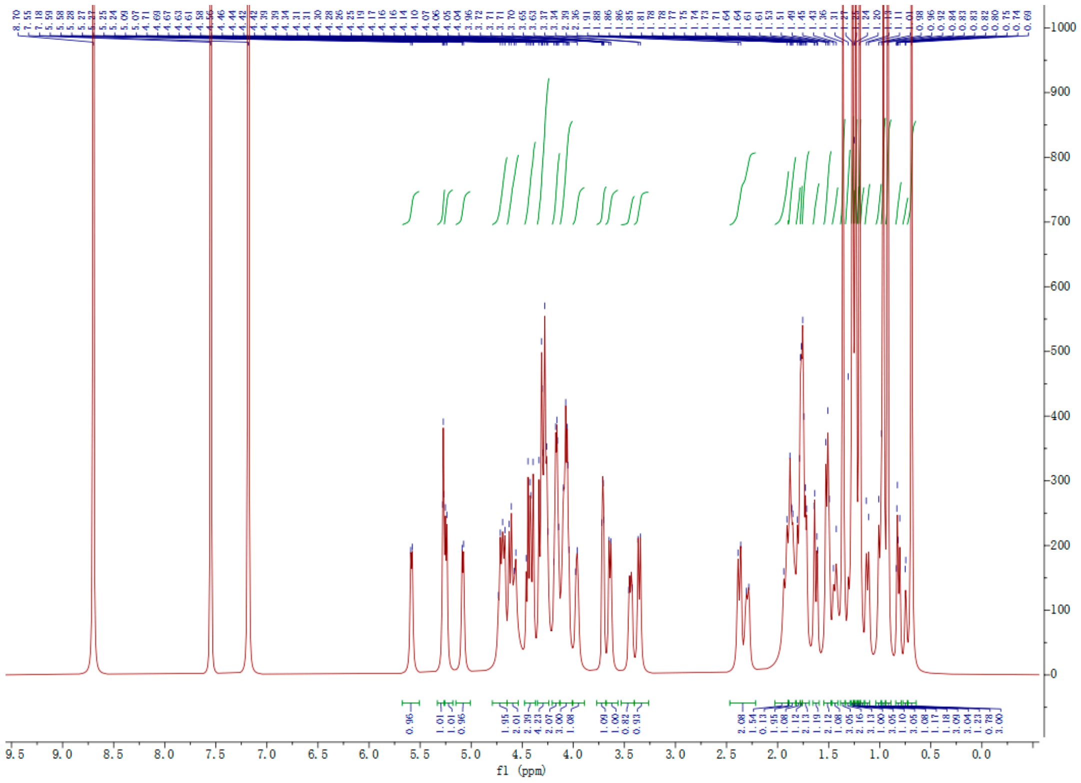

Figure S1. ^{1}H NMR spectrum of compound **1** in C_5D_5N (400 MHz)

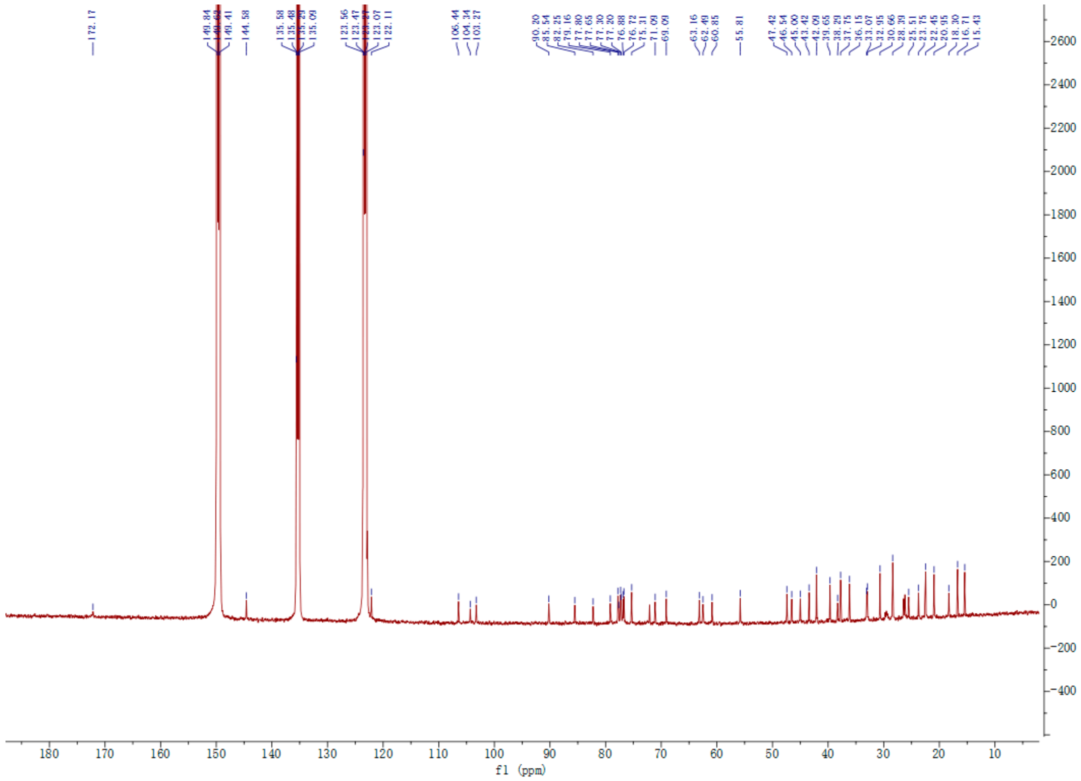

Figure S2. ^{13}C NMR spectrum of compound **1** in C_5D_5N (100 MHz)

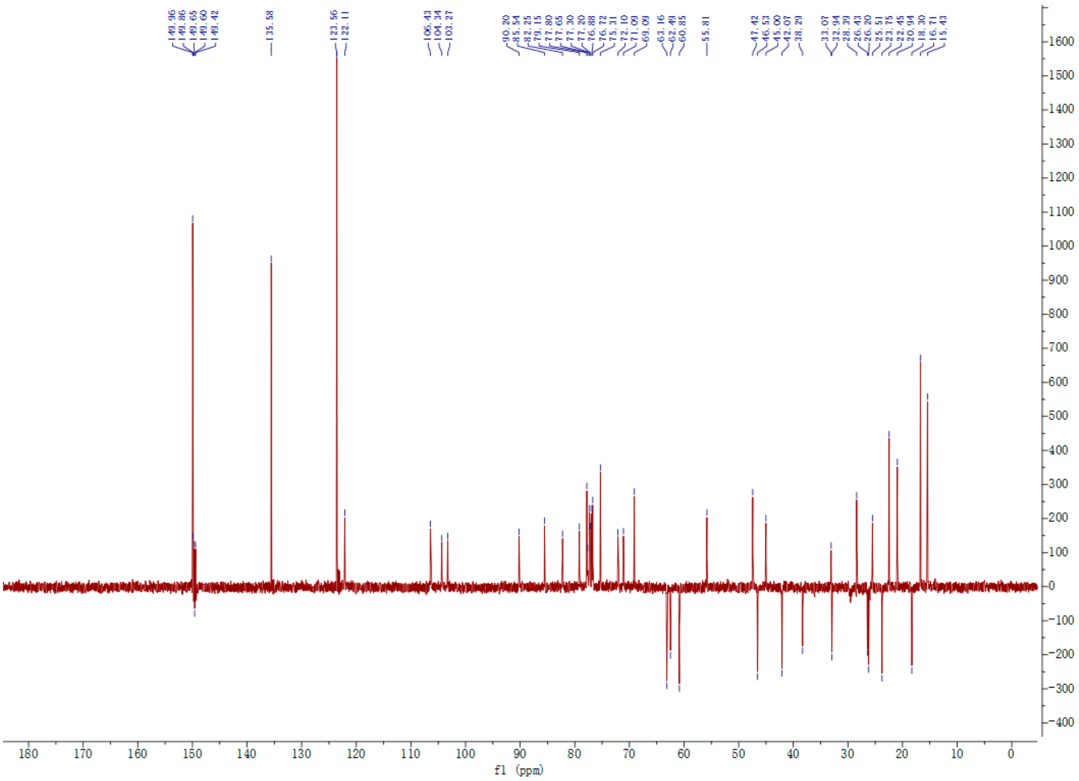

Figure S3. DEPT spectrum of compound **1** in C_5D_5N (400 MHz)

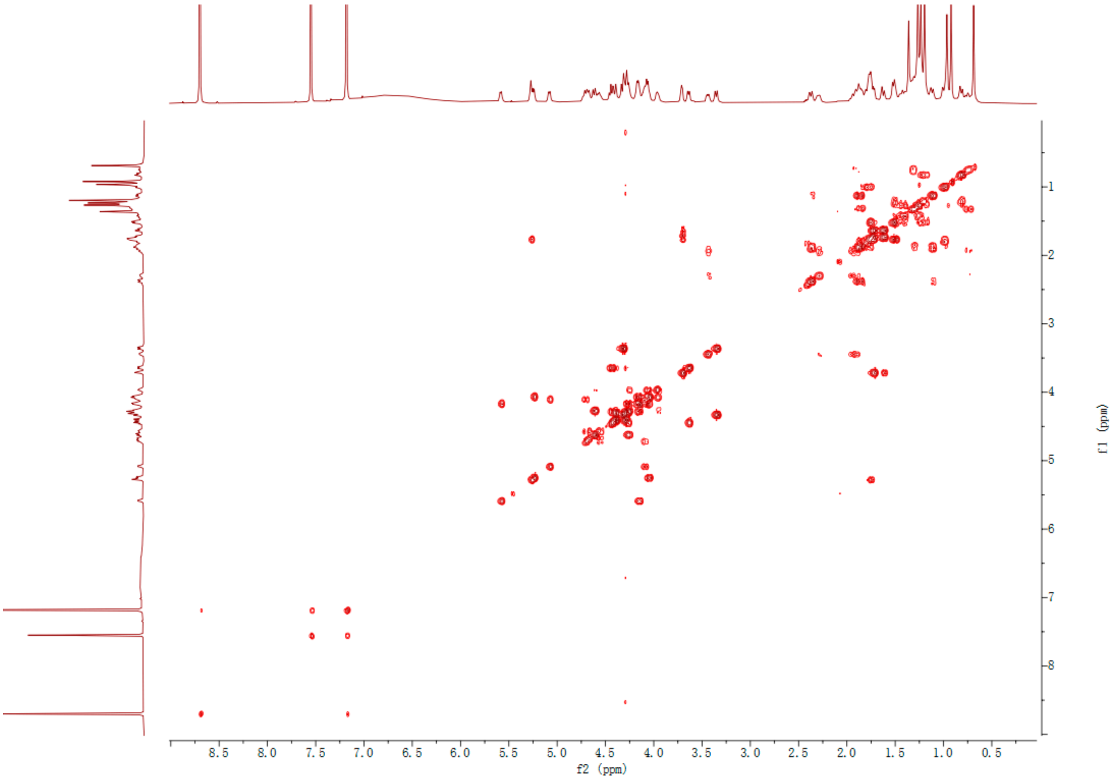

Figure S4. COSY spectrum of compound **1** in C_5D_5N (400 MHz)

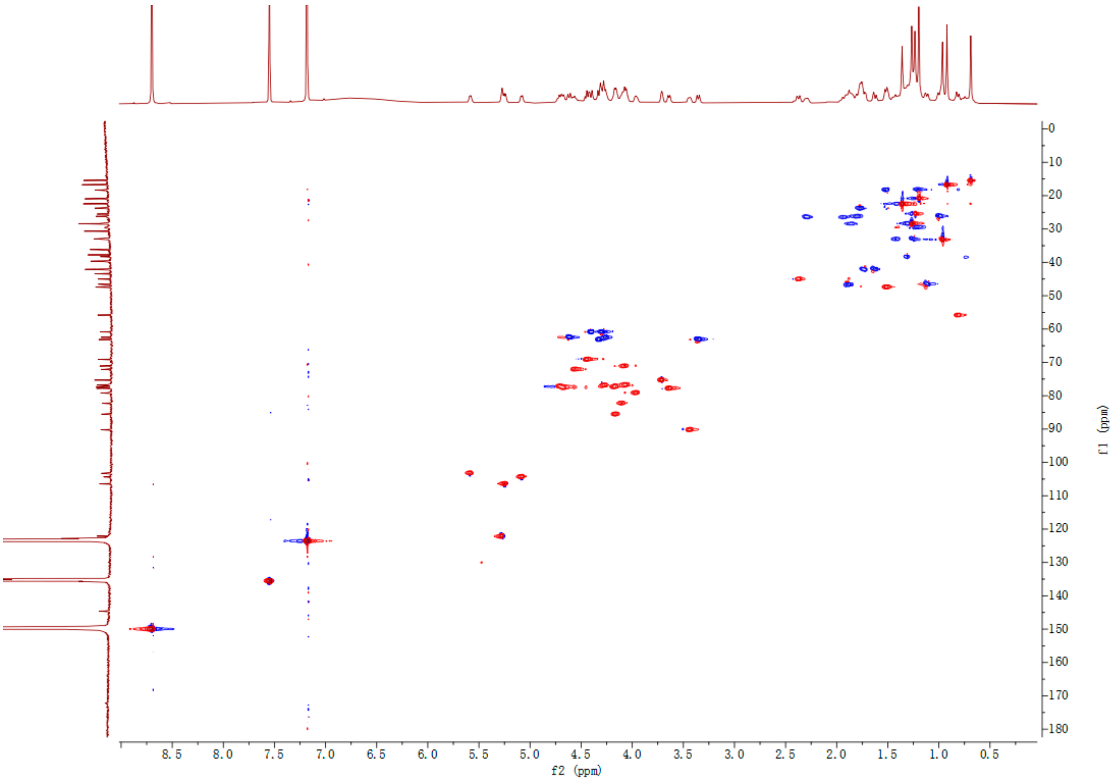

Figure S5. HSQC spectrum of compound **1** in C_5D_5N (400 MHz)

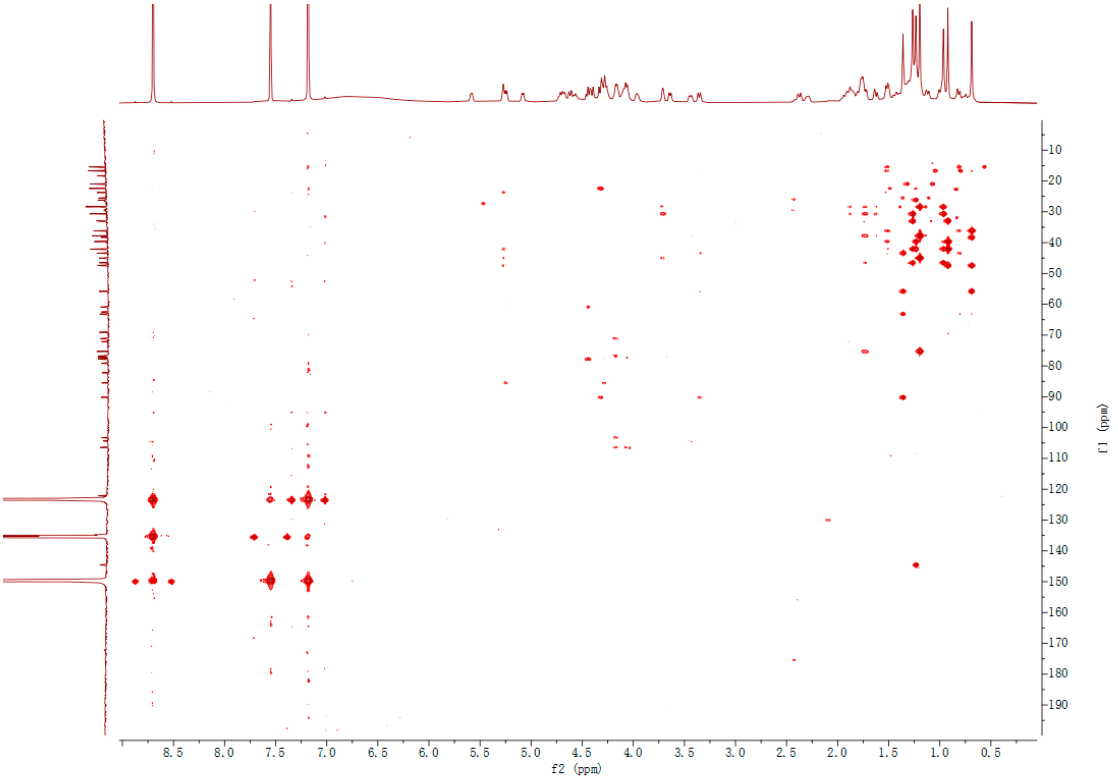

Figure S6. HMBC spectrum of compound **1** in C_5D_5N (400 MHz)

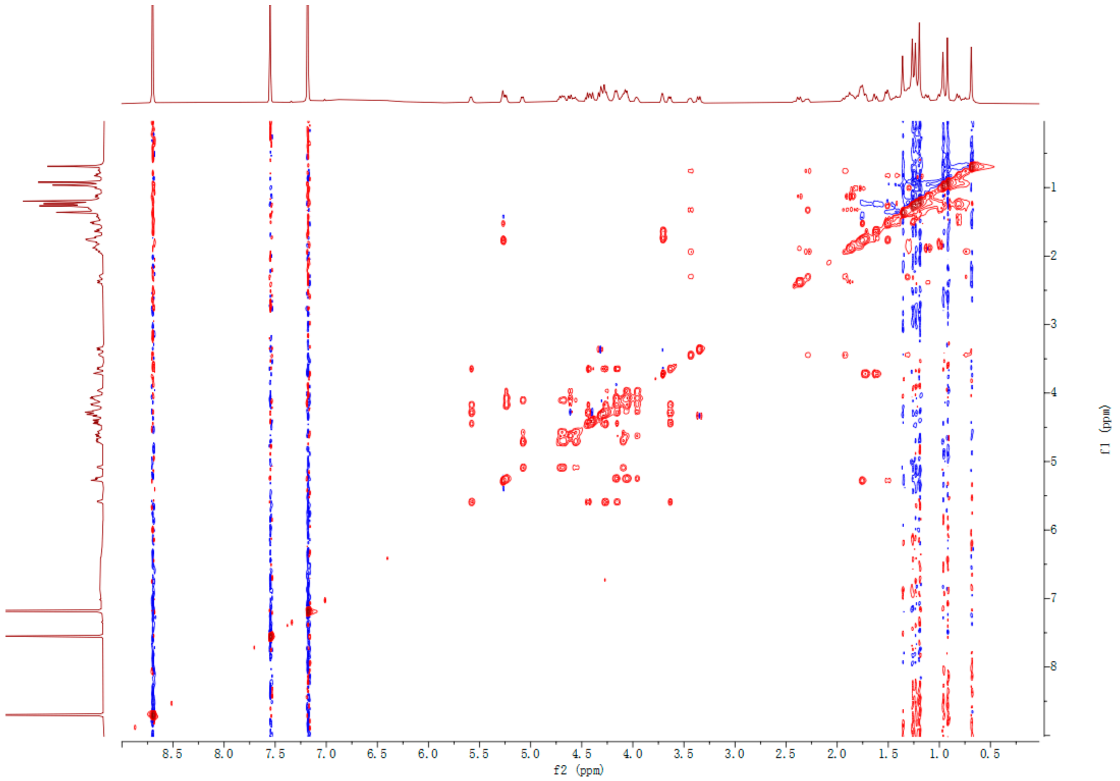

Figure S7. TOCSY spectrum of compound **1** in C_5D_5N (400 MHz)

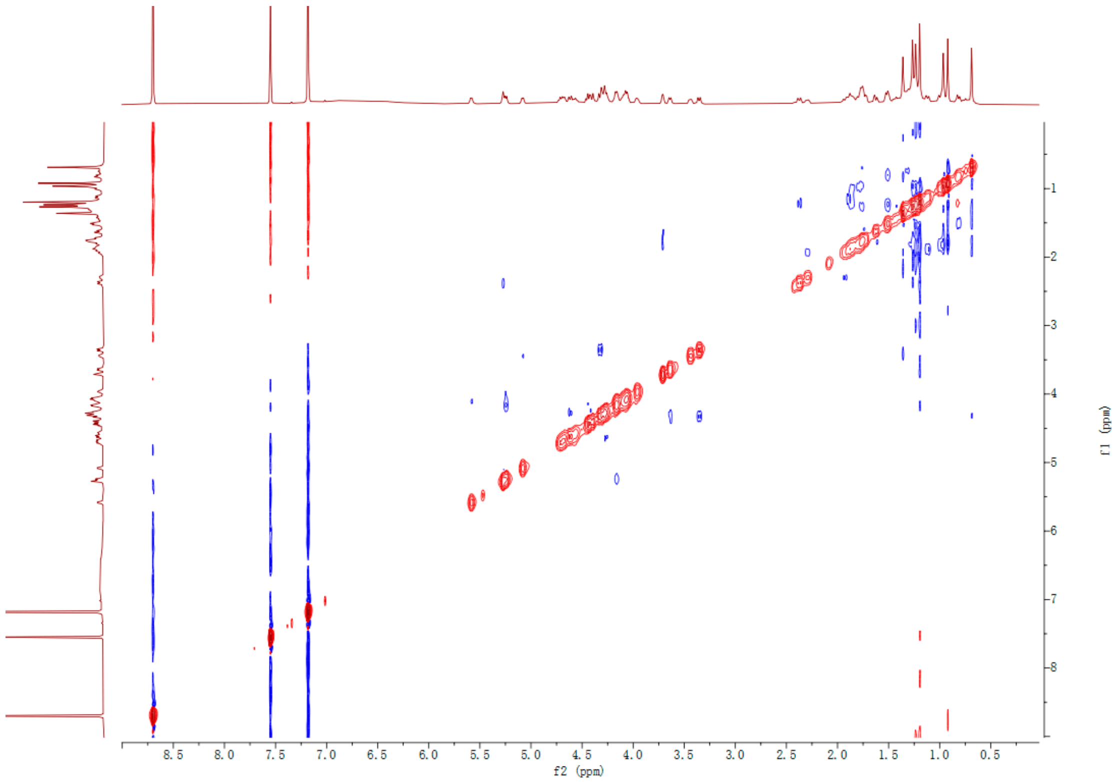

Figure S8. NOESY spectrum of compound **1** in C_5D_5N (400 MHz)

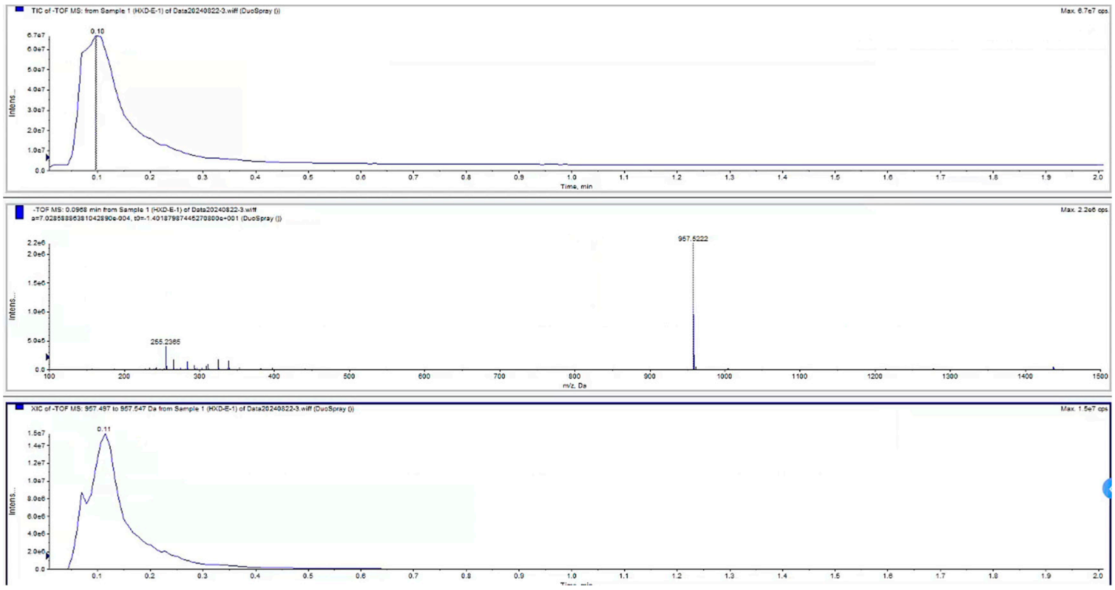

Figure S9. HRESIMS spectrum of compound **1**

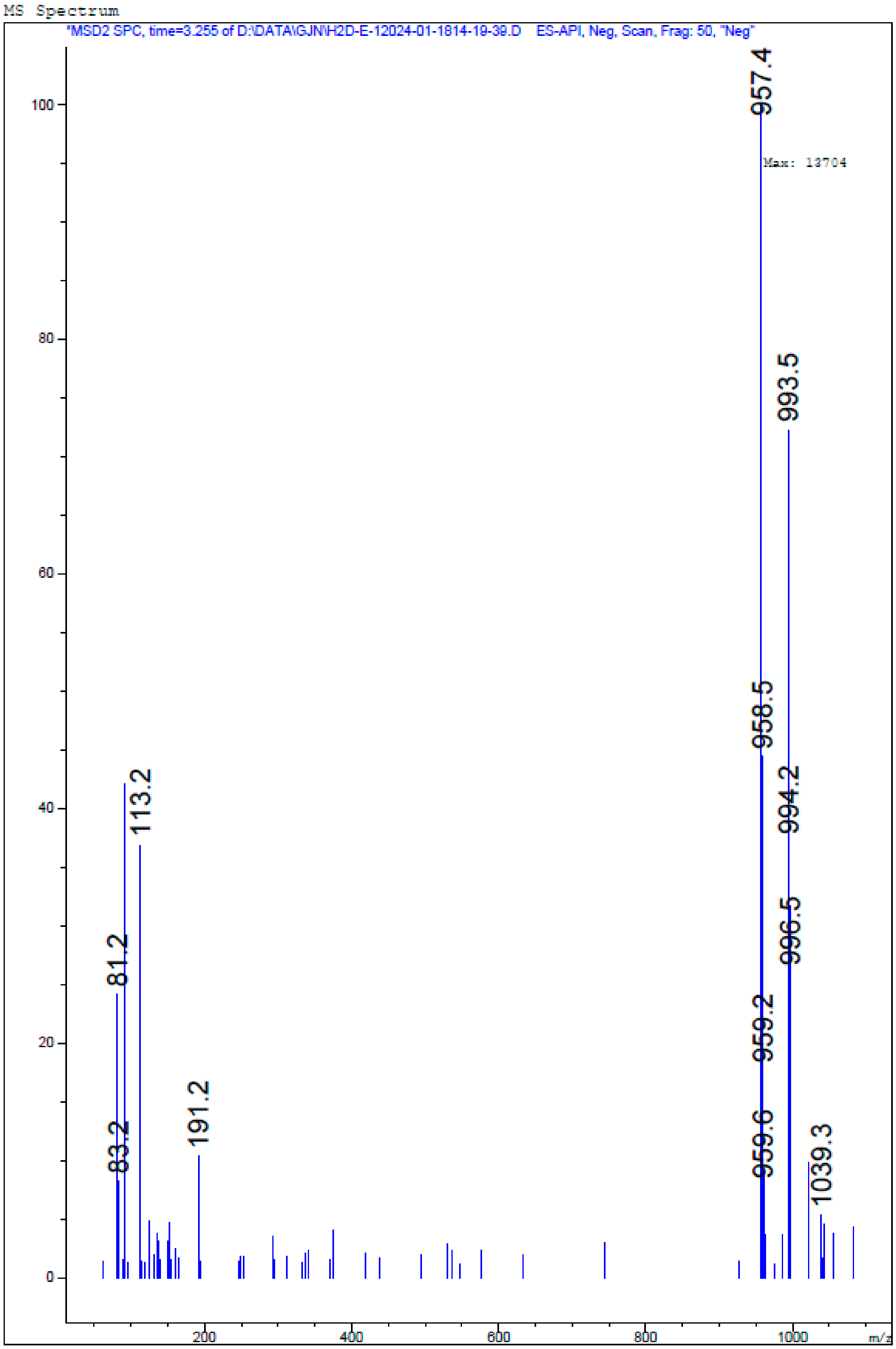

Figure S10. EIMS spectrum of Compound **1**

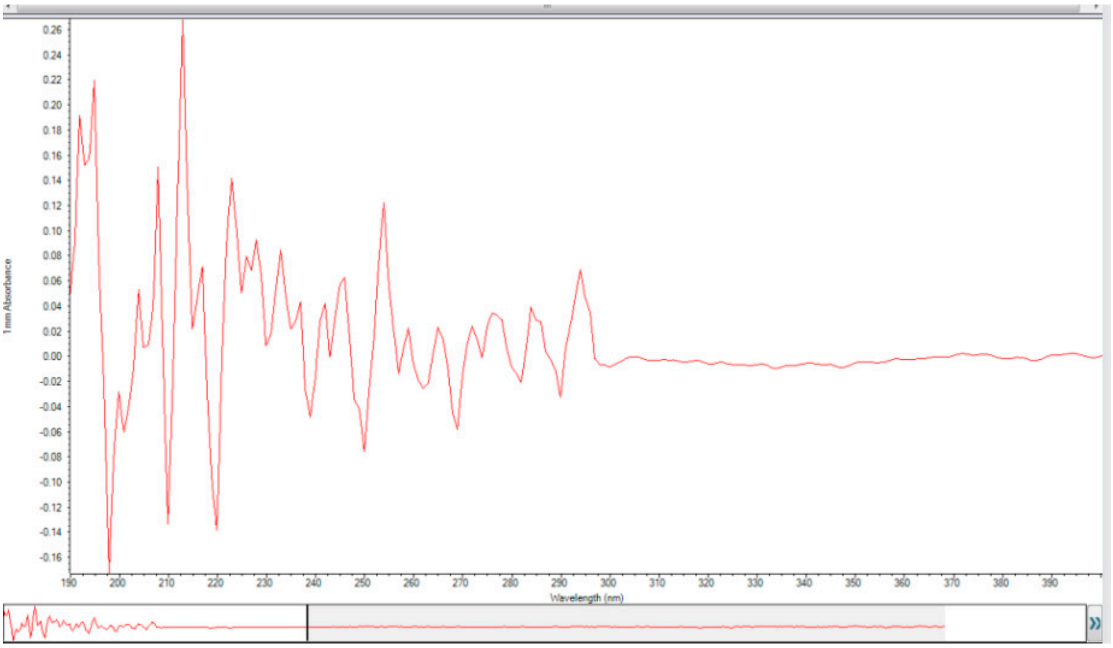

Figure S11. UV spectrum of Compound **1**

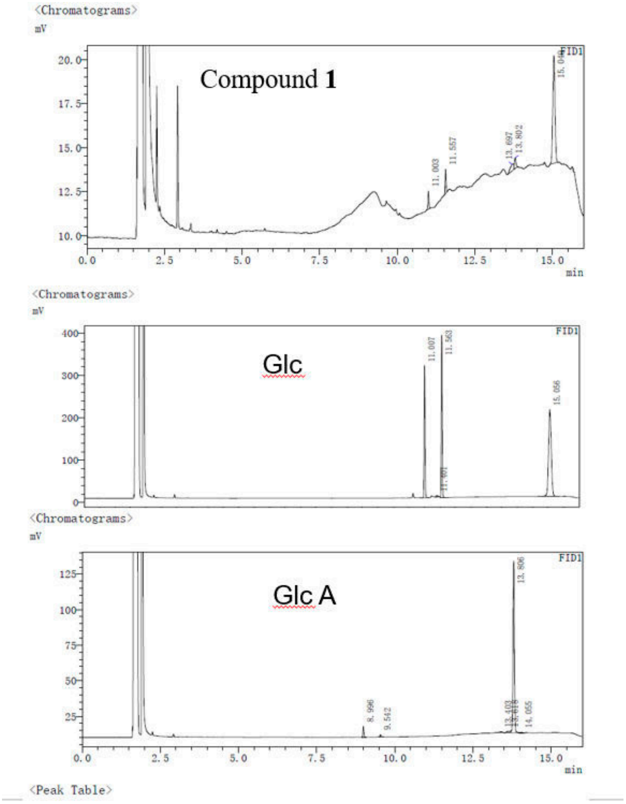

Figure S12. Comparison of the acid hydrolysis products of compound **1** with glucose and glucuronic acid by GC
